# Assessing urban and rural neighborhood characteristics using audit and GIS data: derivation and reliability of constructs

**DOI:** 10.1186/1479-5868-6-44

**Published:** 2009-07-20

**Authors:** Kelly R Evenson, Daniela Sotres-Alvarez, Amy H Herring, Lynne Messer, Barbara A Laraia, Daniel A Rodríguez

**Affiliations:** 1 Department of Epidemiology, Gillings School of Global Public Health, University of North Carolina – Chapel Hill, Chapel Hill, NC, USA; 2 Department of Biostatistics, Gillings School of Global Public Health, University of North Carolina – Chapel Hill, Chapel Hill, NC, USA; 3 Carolina Population Center, University of North Carolina – Chapel Hill, Chapel Hill, NC, USA; 4 Center for Health Policy, Duke Global Health Institute, Duke University, Durham, NC, USA; 5 Department of Medicine, Division of Prevention Sciences, Center for Health and Community, University of California, San Francisco, CA, USA; 6 Department of City and Regional Planning, University of North Carolina – Chapel Hill, Chapel Hill, NC, USA

## Abstract

**Background:**

Measures to assess neighborhood environments are needed to better understand the salient features that may enhance outdoor physical activities, such as walking and bicycling for transport or leisure. The purpose of this study was to derive constructs to describe neighborhoods using both primary (neighborhood audit) and secondary (geographic information systems) data.

**Methods:**

We collected detailed information on 10,770 road segments using an audit and secondary data. The road segment sample was randomly split into an exploratory (60%) and validation sample (40%) for cross-validation. Using the exploratory sample (n = 6,388), seven a priori constructs were assessed separately (functionality, safety, aesthetics, destinations, incivilities, territorality, social spaces) by urbanicity using multi-group confirmatory factor analysis (CFA). Additionally, new a posteriori constructs were derived using exploratory factor analysis (EFA). For cross-validation (n = 4,382), we tested factor loadings, thresholds, correlated errors, and correlations among a posteriori constructs between the two subsamples. Two-week test-retest reliability of the final constructs using a subsample of road segments (n = 464) was examined using Spearman correlation coefficients.

**Results:**

CFA indicated the a priori constructs did not hold in this geographic area, with the exception of physical incivilities. Therefore, we used EFA to derive a four-factor solution on the exploratory sample: arterial or thoroughfare, walkable neighborhood, physical incivilities, and decoration. Using CFA on the validation sample, the internal validity for these a posteriori constructs was high (range 0.43 to 0.73) and the fit was acceptable. Spearman correlations indicated the arterial or thoroughfare factor displayed near perfect reliability in both urban and rural segments (r = 0.96). Both the physical incivilities factor and the walkable neighborhood factor had substantial to near perfect reliability in both urban and rural segments (r = 0.77 to 0.78 and r = 0.79 to 0.82, respectively). The decoration factor displayed moderate reliability in urban segments (r = 0.50; 95% CI: 0.38–0.60) and lower reliability in rural segments (r = 0.39; 95% CI: 0.25–0.52).

**Conclusion:**

The results of our analyses yielded four reliably and objectively measured constructs that will be used to explore associations with physical activity in urban and rural North Carolina. These constructs should be explored in other geographic areas to confirm their usefulness elsewhere.

## Background

Physical inactivity is an important public health issue worldwide [1,2] and there is growing interest on the influence of the environment on physical activity behavior [3]. Using the socio-ecologic framework as a guide, physical activity is influenced by individual, interpersonal, organizational, community or environmental, and public policy or societal characteristics [4,5]. Our focus here is on the development of measures to assess neighborhood environments, to better understand the salient features that may enhance outdoor physical activities, such as walking and bicycling for transport or leisure.

One way to ascertain information about neighborhoods is to solicit self-reported characteristics of neighborhoods from residents or local experts. The challenge with this is that perceptions of the same neighborhood may differ by such factors as gender, age, or socioeconomic status. Furthermore, when study participants self-report neighborhood characteristics and outcomes such as physical activity, the exposure and outcome are subject to same source bias [6]. To study associations between neighborhood environments and physical activity without reliance on self-report, researchers have used existing neighborhood data (e.g., roads, parcels, land uses) in a geographic information system (GIS) to create environmental-based measures.

There are many challenges to using secondary neighborhood data to measure features of the neighborhood environment that may support physical activity [7]. An important challenge is that often only easily collected existing data is used in these analyses, such as from government GIS sources or by review of aerial maps. Yet, secondary data rarely contain the detail necessary to test desirable hypotheses. Relying solely on secondary sources to represent a neighborhood may provide an oversimplified understanding of neighborhoods and may mask within-neighborhood variability that exists [8]. Moreover, data are often non-comparable because they may not have been collected in the same way or during the same time period. There may also be differences in scale, especially for aerial photos. Another challenge is that existing data are rarely able to capture the rapid development or deterioration that characterizes neighborhoods in transition. Furthermore, it is not possible to assess measures such as social interaction within neighborhood by relying on secondary sources only.

Alternatively, researchers have considered neighborhood audits to collect data using observation on a street-by-street basis. In the social sciences, neighborhood audits and systematic social observation protocols represent salient neighborhood characteristics [9,10]. More recently, researchers interested in the relationship of the environment to physical activity considered neighborhood audits designed for research purposes as a potential data source that provides additional information to what is available through secondary data sources [11]. Historically, a number of audit tools were developed to assist communities in making decisions or community members in advocating for changes to pedestrian and bicycling infrastructure [12].

There are several challenges in using neighborhood audits to examine associations with physical activity [13]. The audits generally include many variables, but not much work has been done to create constructs from these individual items. Generally no consideration is given to incorporating secondary data into the constructs. Past audits exploring associations with physical activity also generally have small sample sizes, because the effort to collect this on-the-ground data is substantial. Moreover, neighborhood audits were historically developed for use in urban areas and not conducted in rural areas. It is not known if urban audits will work in rural areas, whether the constructs will operationalize similarly for both areas, and what adaptations might be needed.

The purpose of this study was to derive constructs to describe neighborhoods using both primary data collection, through a neighborhood audit, and secondary data collection. Both databases were brought together in GIS. This work was guided by an a priori framework and was conducted in rural and urban North Carolina, to explore whether the derived constructs were similar in urban and rural environments. We describe a process others can replicate in creating constructs, first testing whether our a priori constructs held in confirmatory factor analysis (CFA) for both urban and rural street segments and if not, using exploratory factor analysis (EFA) to create new constructs.

## Methods

### Study Sample

The third phase of the Pregnancy, Infection, and Nutrition (PIN3) Study recruited 2006 pregnant women at less than 20 weeks' gestation seeking prenatal care at clinics associated with the University of North Carolina Hospitals and followed them to 12 months postpartum [14]. The study website  provides greater detail on the protocols and measures used. Most women lived in central North Carolina, primarily from Alamance, Chatham, Durham, and Orange Counties. These contiguous four counties served as the geographic boundary of the area we studied. Each woman's home address was both collected and geocoded at 27–30 weeks' gestation and at 3- and 12-months postpartum. We selected all road segments within a quarter mile of her home location in order to collect neighborhood audit information. Data collectors were masked to the home address of the participants and also signed confidentiality agreements before working in the field. We also collected detailed existing GIS data from these four counties.

We describe below the a priori framework used for data acquisition, followed by the methodology used for both the primary and secondary data collection and analyses.

### Description of A Priori Constructs

Based on the state of the literature at the time we began this project, we focused on replicating constructs developed by two different research groups. First, we sought to collect items for a neighborhood scale, titled the "Neighborhood Brief Observation Tool" described by Caughy et al [8], consisting of physical incivilities, territorality, and social spaces constructs. We expected that they would be associated with physical activity, although they had not specifically been examined in this way.

**(1) Physical incivilities **was based on the work by Perkins et al [15], defined as physical disorder that is associated with increased crime, including items such as trash and vacant buildings.

**(2) Territorality**, derived from two constructs (territorial functioning and defensible spaces), was based on the work of Taylor et al [16,17]. Territorial functioning includes markers which convey a non-verbal message about control, separation from outsiders, and investment in the neighborhood. Indicators included property maintenance, decoration, and symbols of protection. Defensible spaces included characteristics of the neighborhood that encourage residents to exercise territorial control, such as fences or borders. Similar to Caughy et al [8], we proposed these two constructs together because in prior literature they were strongly related to each other.

**(3) Social spaces **was developed in follow-up work by Laraia et al [18], expanding the original availability of play resources scale by Caughy et al [8] to be relevant for both children and adults and included items such as presence of yard, parks, and physically active people.

Second, for the constructs directly related to physical activity in and around the neighborhood, we sought to collect data on constructs developed from the Pikora et al [19] conceptual framework for walking and bicycling. The physical environmental features and elements proposed to be associated with walking and bicycling included four constructs:

**(4) Safety **included both personal, such as presence of lighting and sidewalk buffer, and traffic, such as crosswalks. These two elements were combined in the analysis.

**(5) Aesthetics **related to the interesting and pleasing physical environment for walking or bicycling. This construct included two elements: streetscape (e.g., trees, porches, and decoration) and views (e.g., abandoned residential or nonresidential units, vacant land, or industrial land).

**(6) Functionality **related to physical attributes of the street. This construct included four elements: surface (e.g., road surface type, maintenance, and continuity), streets (e.g., width of road), traffic (e.g., volume, speed, and traffic control devices), and permeability (e.g., intersection design and distance).

**(7) Destinations **referred to places to walk or bike to and included parks and bus stops. We expanded the items included to include religious structures and home based businesses.

### PIN3 Neighborhood Audit Instrument Development

**Figure 1:**
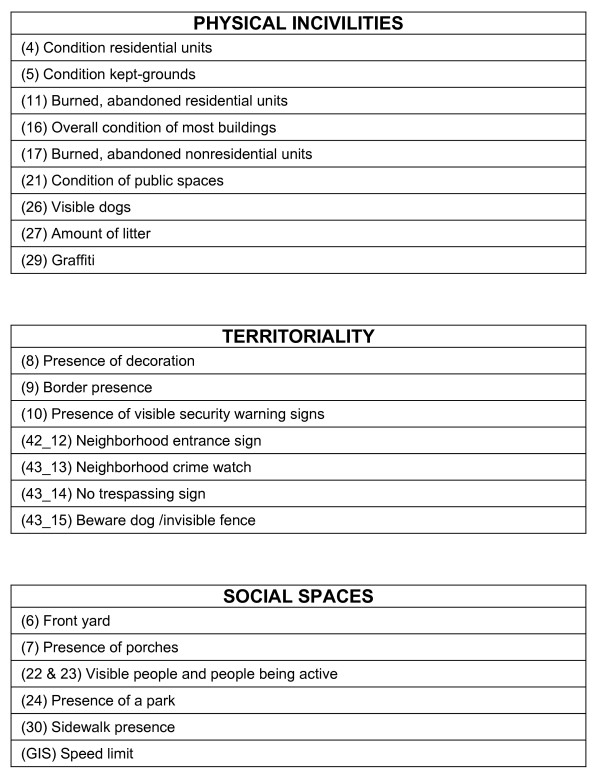
**A priori constructs to assess the neighborhood environment from Caughy et al (2001), with corresponding items from the PIN3 Neighborhood Audit instrument (item numbers specified in parentheses) or from GIS measures**.

**Figure 2:**
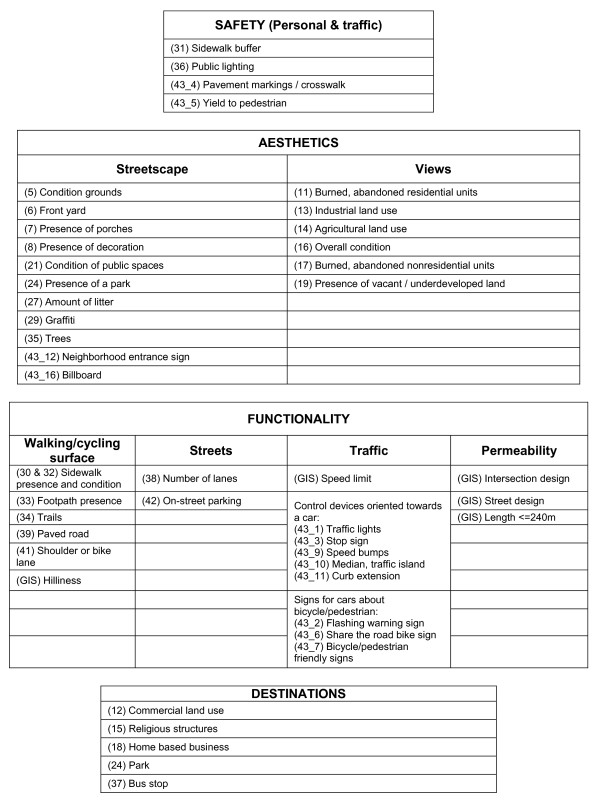
**A priori constructs to assess the neighborhood environment from Pikora et al (2002), with corresponding items from the PIN3 Neighborhood Audit instrument (item numbers specified in parentheses) or from GIS measures**.

We developed our PIN3 neighborhood audit instrument to collect data not available in the four county study area from secondary data and to contribute to the derivation of the a priori constructs described. A candidate list of audit items was extensively pilot tested in the local area. Some items were modified in order to capture variation in the counties under study and to enhance relevancy to the area, since the original instruments were developed for use in Baltimore, Maryland (United States or US) and in Perth (Australia), respectively [8,19]. The final list of items, along with their mapping to each construct, is shown in Figures 1 and 2. We allowed some items to load on more than one a priori construct. The paper version of the PIN3 Neighborhood Audit instrument can be found elsewhere [see Additional file 1]. This version was adapted for data collection using handheld electronic devices.

### Data Collection Procedures

The four-county study area covered approximately 1,843 square miles with 7,150 miles of roads. In summer of 2005 and 2006, our team assessed 10,770 street segments out of a possible 41,683 street segments (25.8%) in this area. A street segment was defined as the road length between two intersections or between an intersection to a cul-da-sac or dead end road. Extensive work went in to creating an accurate road database [20]. Each road segment was assigned a unique identifier and selected for rating if it fell within one-fourth mile of each participant's home address. From this database, maps were created and printed for the raters to use while out in the field.

Each rater completed a week-long training that included classroom and field-based training; reliability testing was conducted until raters reached an acceptable level of agreement. In total, 10 raters were trained in 2005 and 6 were trained in 2006. The raters worked in pairs and assessed street segments by driving slowly up and down the segment several times and then pulling over to complete the assessment tool on a handheld device (Palm Pilot). They rated the road segments between 9 am to 4 pm on weekdays and did not rate during bad weather. Teams completed approximately 5 street segments per hour, which included driving time to the various segments.

To help maintain reliability of measurement over time, raters rotated partners, regular team meetings were held for questions and review, and reliability was assessed every two to three weeks. Results from this testing were discussed at team meetings to help identify any discrepancies in rating between reviewers. Additionally, some of the same raters from 2005 rated in 2006, which helped maintain consistency over time. Two-week test retest reliability (mean 15.5 days, median 16 days, interquartile range 10–20 days) was conducted on 464 road segments in 2005 and is reported herein. Different teams rated the same road segment within this time period, such that no person ever rated the same segment twice.

### Secondary Neighborhood Data

We considered five variables (cul-de-sac or dead-end road, 3- or 4-way intersections, road segment length, speed limits, slope) for each road segment in the 4-county area from our customized road network database [20]. We describe each briefly, with further information elsewhere [21]. Using GIS, we determined whether or not a road segment was a cul-de-sac or dead-end road and a 3- or 4-way intersection. Each road segment length was determined from the road database. Speed limits for each road segment were assigned according to type of road (e.g., interstates, secondary road, neighborhood/subdivision) and in some cases whether or not the segment was in an urban area. Speed limit was collapsed into three levels based on the distribution of the data (< = 25 mph, 26-< = 45 mph, >45 mph). Although we collected speed limit from the neighborhood audit when posted, we chose not to use that variable since further assumptions were needed to assign speed limits to roads without signage. The Spearman correlation between speed limits collected by the audit compared to speed limits collected using GIS was 0.68 (n = 3121). The agreement among segments was κ = 0.43 (95% CI 0.40, 0.47) and κ = 0.55 (95% CI 0.52, 0.58) for rural and urban segments, respectively. To determine slope, each road segment was divided into 100 foot sections and the slope of that section was extracted from a digital elevation model of the study area with 100 feet resolution. We created a 2-level variable defined as half or more of the street segment with >5% slope. We also explored cutpoints at >3% and >8% slope and found no differences in results.

To define road segments as either urban or rural, we used the US Census Bureau classification of urban areas, defined as (1) core census block groups that have a population density of at least 1,000 people per square mile and (2) surrounding census block groups that have an overall density of at least 500 people per square mile [22]. In addition, under certain conditions, less densely settled territory may be part of each urbanized area or urban cluster. The Census Bureau's classification of rural consists of all territory, population, and housing units located outside of urbanized areas and urban clusters. We used the census data to define urbanicity for each street segment. If the midpoint of the segment fell within an urban block group, then a segment was categorized as urban and otherwise as rural.

### Statistical Analysis

The hypothesized a priori constructs as well as those derived a posteriori were based on the PIN3 Neighborhood Audit instrument [see Additional file 1] and secondary (county government GIS data. Some categorical variables were collapsed when cell sizes were sparse or to make them ordinal. Several audit variables were combined into composite items since they were dependent on each other or were sparsely observed:

- visible and active people (#22 visible people and #23 people being active, separately for youth and adults);

- sidewalk condition (#30 presence of sidewalk and #32 condition);

- control devices oriented towards a car (#43_1 presence of traffic lights, #43_3 stop signs, #43_9 speed bumps, #43_10 median/traffic islands, and #43_11 curb extensions); and

- control devices or signs about pedestrians or bicyclists (#43_2 flashing warning sign, #43_6 "share the road" bicycle sign, and #43_7 other pedestrian or bike friendly traffic signs).

The road segment sample was randomly split into an exploratory sample (60% of the street segments rated) and a validation sample (40% of the street segments rated) to conduct cross-validation. Descriptive analyses include frequencies for audit and GIS variables, and standardized Cronbach's alpha for a priori and a posteriori constructs. We accounted for correlation between segments within neighborhoods by clustering segments in block groups and using complex survey data commands. All analyses were frequency weighted by the street segment's length because of the range in road lengths.

Using the exploratory sample (n = 6,388), seven hypothesized constructs [8,11] were assessed separately using multi-group confirmatory factor models to test measurement invariance between rural and urban segments (i.e., we tested if parameters were the same in rural and urban segments). In contrast to EFA in which the relationship between the observed and the factors is not specified in advance, in CFA a statistical model that attempts to explain the correlations between many observed variables by few underlying but unobservable variables called factors is specified a priori and hence, some factor loadings are restricted to zero when specific items do not load on respective factors [23,24]. Because factors or constructs are unobserved, their mean was constrained to zero and their scale was set to the scale of one of the observed variables for identifiability (i.e., unique solution). The factor loadings were estimated using weighted least squares and the factors were derived to be orthogonal (i.e., uncorrelated) using the Varimax rotation. This type of rotation makes the factors' interpretation easier, since the factor loadings are more extreme by being closer to 0 or ± 1 rather than being intermediate (around 0.5). In order to assess the goodness-of-fit, we used three measures: root mean square error of approximation (RMSEA), comparative fit index (CFI), and Tucker-Lewis index (TLI). The RMSEA is a stand-alone goodness-of-fit, whereas the CFI and the TLI compare the fitted model to the independence model (e.g., no factors underlie the observed variables and the correlations between the observed indicators are zero). Guidelines for lack of fit are given empirically by rules of thumb or some are based on simulations [25]. In practice, the CFI or TLI above 0.95 and the RMSEA below 0.05 are considered a good fit.

Because several of the hypothesized constructs had poor goodness-of-fit, we derived new constructs a posteriori using EFA. Because there is no best and single criterion as to how to determine the number of factors, we used the Cattel's Scree plot criterion (where the plot abruptly levels out) on the eigenvalues of the correlation matrix, as well as the factors' interpretability by visual inspection of the loadings [24]. First, the structure of the constructs was identified from the items with exploratory factor loadings ≥ 0.4. Items could load on several factors. Due to sparse cell counts, some residual variances were negative and hence, these items were dropped from the EFA to obtain an admissible solution. Second, to assess goodness-of-fit, we initially fit 1-factor confirmatory models separately by urbanicity for each derived construct, including those dropped variables that loaded on the other group (rural, urban) to contain the same variables. In order to improve the goodness-of-fit, models were slightly modified by removing an item or adding correlated errors based on the modification indices (e.g., improvement in goodness-of-fit chi square when comparing models) and interpretability. Third, we fit a multiple factor model by urbanicity and allowed the factors to be correlated in order to test if, after simplification (e.g., constraining some of the factor loadings to zero), they were still orthogonal. The name of the constructs, although subjective, was given according to the items with higher factor loadings.

For cross-validation (n = 4,382), we tested that factor loadings, thresholds, correlated errors and correlations among a posteriori constructs were the same between the two subsamples. Finally, two-week test-retest reliability of the final constructs was examined using Spearman correlation coefficients. As a rough guide, we followed the ratings suggested by Landis and Koch [26] for agreement level: 0–0.2 poor, 0.2–0.4 fair, 0.4–0.6 moderate, 0.6–0.8 substantial, and 0.8–1.0 almost perfect. All statistical analyses were performed using Mplus, Version 5.1 (Muthén and Muthén, 1998–2007) or SAS software, Version 9.1 of the SAS System for Windows (SAS, 2002–2003, Cary, NC).

## Results

### Descriptive Statistics

Of the 10,770 road segments rated, 7,660 (71.1%) were in urban areas and 3,110 (28.9%) in rural areas. After weighting for road segment length, this corresponded to 61.3% and 38.7%, respectively. Urban segments were generally shorter than rural segments. Half of the urban road segments were less than 134 m long (interquartile range: 92–212 m) and 5% were longer than 420 m. In contrast, in rural areas the median road segment length was 173.5 m (interquartile range: 102–316 m) and 5% were longer than 849 m.

The frequency and weighted percent (weighted for road length) for the GIS and audit items overall and by urbanicity for the entire audit sample (n = 10,770) are shown elsewhere [see Additional file 2]. The numbering in the table corresponds to the items used from the PIN3 Neighborhood Audit instrument [see Additional file 2]. Compared to urban road segments, rural road segments were more often cul-de-sacs, longer in length (> = 240 m), and had higher speed limits, but less often had 3- or 4-way intersections. More rural road segments (50.7%) had no visible security warning signs on the road segment as compared to urban segments (34.3%). Nineteen percent of rural road segments included agricultural land, compared to only 0.8% on urban road segments. Urban road segments had 3 times more segments with sidewalks than rural road segments (38.4% and 11.6% respectively). Any road oriented lighting along the street was much more common on urban segments (71.2%) as compared to rural segments (23.1%).

### Exploratory Factor Analysis on A Priori Constructs

Several items on the PIN3 neighborhood audit were not considered in our factor analysis. We did not consider the following variables due to their nominal coding and in some cases narrow distribution: type of housing (#3), overall condition of most buildings (#16), condition of vacant land (#20), park condition (#25), type of litter (#28), bicycle parking (#43_8), and billboards (#43_16). We used a GIS-derived speed limit measure, as described earlier, rather than our audit assessed measure (#40) since many segments did not have a speed limit sign posted. We also used the item on the number of residential units (#2) only as an indicator of whether the rest of the section on residential housing was answered. Lastly, the subjective assessment (#1) was an indicator of the rater's assessment and was not intended to represent any of the a priori constructs and therefore not used.

Standardized Cronbach's alphas are presented in Table 1 for all a priori constructs. Physical incivilities had the highest internal consistency and its consistency was similar among urban and rural segments (alpha = 0.57 and 0.59, respectively). All other constructs had lower internal consistency (range 0.11 to 0.44). Territoriality and aesthetic views had higher consistency among rural segments, whereas social spaces and destinations had higher consistency among urban segments. Safety, aesthetic streetscape, and functionality had similar internal consistency by urbanicity.

**Table 1:** Standardized Cronbach's alpha by urbanicity and multi-group one-factor analysis^§ ^for *a priori *constructs, exploratory sample (n = 6,388).

**Construct**		Standardized Cronbach's Alpha^§^		Goodness-of-fit for non-invariant model				Difference test^‡^
					
		Urban	Rural	Number of parameters	CFI > 0.95*	TLI > 0.95*	RMSEA < 0.05*	p-value
**Constructs from Caughy et al, 2001**								
**Physical incivilities**		0.57	0.59	40	0.960	0.951	0.002	<0.0001
**Territoriality**		0.22	0.34	34	0.211	0.096	0.005	<0.0001
**Social spaces**		0.31	0.24	38	0.664	0.614	0.042	<0.0001

**Constructs from Pikora et al, 2002**								
**Safety**		0.39	0.41	24	0.774	0.661	0.006	<0.0001

**Aesthetics**	**Streetscape**	0.29	0.27	52	0.001	0.287	0.006	<0.0001
	**Views**	0.28	0.44	20	1	1	0	0.983

**Functionality**	**All items together**	0.18	0.19	70	0.791	0.791	0.004	<0.0001

**Destinations**		0.27	0.11	19	0.203	0.595	0.011	<0.0001

§ Data are weighted by the road segment's length.

‡ Ho: Invariance (i.e. all factor loadings and thresholds are the same by urbanicity) H_1_: Non invariance (i.e. different factorloadings and thresholds by urbanicity)

Abbreviations: RMSEA root mean square error of approximation, CFI comparative fit index, and TLI Tucker-Lewis index

*These are "rules of thumb" guidelines to interpret these indices (see paper for more information).

Table 1 also presents the invariance test (e.g., same factor loadings and thresholds or not by urbanicity) for 1-factor models and their goodness-of-fit for non-invariant models. For all a priori constructs, except for aesthetic views, the invariance test was highly significant indicating that the measures were not the same between urban and rural segments. However, the variability of the aesthetic views and destinations constructs were not significantly different from zero, and hence these constructs were not useful. Further, non-invariant models had poor fit except for physical incivilities and, hence this data did not support these hypothesized models for the remaining constructs. The second-order confirmatory factor model for functionality with four elements (walking/bicycling surface, streets, traffic, permeability) did not converge due to negative factor variances for traffic and permeability, and hence we simplified functionality to one factor explaining all items simultaneously. We also explored the functionality construct analyzing the four components separately (e.g., walking/bicycling surface, streets, traffic, permeability). Internal consistencies were low and factors fit poorly (data not shown).

Table 2 presents the standardized and unstandardized factor loadings for the partial-invariant model (some factor loadings and thresholds constrained to be the same) of physical incivilities, which was the only construct that had an improved model fit compared to the non-invariant model. In rural road segments, litter was the item that was best explained by the physical incivilities factor (R^2 ^= 0.47) and general condition of public spaces was the least explained (R^2 ^= 0.19). In urban road segments, the variability of physical incivilities explained by overall condition of units, grounds, and public spaces was much higher. Overall condition of most residential units and resident-kept grounds were best explained (R^2 ^= 0.71 and 0.74, respectively). The loading for visible dogs was not significantly different from zero in urban segments. Overall, the variability of physical incivilities in urban segments was twice that in rural segments.

**Table 2:** Unstandardized and standardized factor loadings for physical incivilities by urbanicity, using the exploratory sample (n = 6,388)

		**Unstandardized**		**Standardized**		**R^2^**	
				
**Item**	**Description**	**Urban**	**Rural**	**Urban**	**Rural**	**Urban**	**Rural**
4	Overall condition of most residential units	1	1	0.84	0.63	0.71	0.39
5	Overall condition of resident-kept grounds	1.02	1.02	0.86	0.64	0.74	0.41
11	Burned, abandoned residential units	0.78*	1.02*	0.66	0.64	0.43	0.41
17	Burned, abandoned nonresidential units	0.64*	0.93*	0.54	0.58	0.29	0.34
21	General condition of public spaces	0.69	0.69	0.58	0.43	0.34	0.19
26	Visible dogs	0.00*	0.73*	-	0.46	-	0.21
27	Amount of litter	0.80*	1.10*	0.67	0.69	0.45	0.47
29	Presence of graffiti	0.22*	0.85*	0.19	0.53	0.04	0.28
							
Factor mean		0	0	0	0		
Factor variance		0.707	0.392	1	1		

* Unstandardized factor loadings are simultaneously significantly different (p < 0.05) between urban and rural segments.

‡ Ho:Partial invariance (i.e. same factor loadings and thresholds constrained to be the same)

H1: Non invariance (i.e. different factor loadings and thresholds by urbanicity)

### Exploratory and Confirmatory Factor Analysis on New Constructs

Given that not all a priori constructs held, we decided to conduct an EFA with all audit and GIS variables to derive, by urbanicity, orthogonal constructs suggested by the data. This was done using the exploratory sample (n = 4,553 urban and n = 1,846 rural segments). According to the Scree plot criterion, four factors emerged for rural road segments and three factors for urban road segments. However, we selected a four-factor solution for urban segments (rather than a 3-factor solution as indicated from the Scree plots), since from visual inspection of the factor loadings for decoration, the fourth factor was similar to the one identified for rural segments. Table 3 presents the exploratory factor loadings ≥ 0.4 for the 4-factor exploratory solution for urban and rural road segments.

**Table 3:** Exploratory and confirmatory factor loadings^† ^for four-factor models by urbanicity, for the exploratory sample (n = 6,388)

	**Arterial or Thoroughfare**				**Walkable Neighborhood**				**Physical Incivilities**				**Decoration**			
				
(Item #)* Description	**Urban**		**Rural**		**Urban**		**Rural**		**Urban**		**Rural**		**Urban**		**Rural**	
								
	**EFA**	**4-CFA**	**EFA**	**4-CFA**	**EFA**	**4-CFA**	**EFA**	**4-CFA**	**EFA**	**4-CFA**	**EFA**	**4-CFA**	**EFA**	**4-CFA**	**EFA**	**4-CFA**
(GIS) Cul-de-sac	-0.65	-0.60	-0.76	-0.74												
(GIS) 3-/4-way intersection	0.38	0.35	0.44	0.43												
(GIS) High speed limit	0.70	0.81	NI	0.78												
(4) Poor condition of residential units									0.89	0.93	0.74	0.89				
(5) Poor condition of residential grounds									0.80	0.79	0.55	0.71				
(7) Presence of porches													0.44	0.57	0.67	0.56
(8) Presence of decoration													0.54	0.98	0.85	0.99
(9) Presence of border													0.47	0.23	0.33	0.22
(11) Presence of abandoned residential units									0.60	0.65	0.62	0.57				
(12) Presence of commercial use	0.52	0.60	0.36	0.44												
(15) Presence of religious structures	0.44	0.48	0.12	0.00												
(17) Presence of burned/abandoned nonresidential units									0.32	0.53	0.60	0.45				
(21) Condition of public spaces not excellent									0.55	0.47	0.33	0.51				
(22&23) Visible/active child/youth					0.51	0.49	0.37	0.38								
(22&23) Visible/active adult					0.51	0.41	0.46	0.15								
(24) Presence of neighborhood park or playground					0.87	0.83	0.53	0.33								
(26) Presence of dogs									0.14	0.00	0.39	0.33				
(27) Amount of litter									0.56	0.72	0.73	0.62				
(31) Wider sidewalk buffer	0.60	0.46	0.45	0.44	0.39	0.39	0.65	0.76								
(30&32) Sidewalk in good condition	0.61	0.48	0.40	0.52	0.53	0.62	0.73	0.85								
(34) Presence of trails					0.48	0.41	0.18	0.00								
(36) Pedestrian oriented lighting					0.55	0.62	0.49	0.53	-0.63	-0.47	-0.73	-0.58				
(37) Presence of bus facilities	0.64	0.69	NI	0.00												
(38) Many lanes to cross	0.74	0.82	0.84	0.93												
(39) Paved road	0.49	0.39	0.79	0.79												
(41) Shoulder or bike lane	0.47	0.64	0.71	0.62												
(42) On-street parking allowed					0.42	0.35	0.54	0.76								
(43_4) Pavement markings, crosswalk	0.85	0.82	NI	0.62												
(43_5) Yield to pedestrian paddles, signal, crossing street sign	0.77	0.78	0.35	0.56	0.28	0.38	0.60	0.48								
(43_12) Neighborhood entrance sign					0.74	0.80	0.45	0.50								
(43_14) No trespassing sign									0.42	0.34	0.63	0.52				
(43††) Control devices oriented for cars					0.46	0.47	0.45	0.25								

† Fifteen items with EFA factor loadings < 0.4 were excluded from the table: short segment (GIS), steep segment (GIS), type of front yard (#6), security warning sign (#10), industrial land (#13), agricultural land (#14), home business (#18), vacant or underdeveloped land (#19), graffiti (#29), footpath (#33), trees (#35), road oriented lighting (#36), neighborhood crime watch (#43_13), beware of dog or invisible fence signs (#43_15), and signs for cars regarding bike/pedestrian (combined index from #43_2, #43_6, and #43_7).

NI Not included because residual variances were negative due to small cell count.

The eigenvalues of the correlation matrix for the EFA in urban segments were 7.3, 5.6, 4.2, and 2.9 and in rural segments were 7.4, 5.1, 3.7, and 3.4 for arterial or thoroughfare, walkable neighborhood, physical incivilities, and decoration, respectively.

Correlated errors: cul-de-sac with speed; visible/active child/youth with visible/active audlts; sidewalk buffer with sidewalk condition.

†† Derived as a count for presence of traffic lights, stop signs, speed bumps, median/traffic islands, and curb extensions (#43_1, 43_3, 43_9, 43_10, and 43_11).

*The item number corresponds to the question number in supplementary file #1.

One factor loaded very high on higher speed limit, several lanes to cross, and pavement markings. It also loaded on 3- and 4-way intersections, paved road, presence of commercial uses, and presence of a shoulder or bike lane. We called this factor "**arterial or thoroughfare**". Factor loadings were in the same direction for urban and rural segments, although of different magnitude. Three items (e.g., high speed, bus facilities, and pavement markings) were excluded from the EFA for rural segments due to sparse cell sizes.

A second factor loaded high on visible children and adults, presence of a neighborhood park or playground, neighborhood entrance signs, lighting oriented for pedestrians, and control devices oriented for cars. We called this factor "**walkable neighborhood**".

A third factor identified was very similar to the a priori physical incivilities construct. It loaded on poor condition of residential units, poor condition of grounds, abandoned units, presence of litter, presence of dogs, no trespassing signs, and absence of pedestrian-oriented lighting. This factor was called "**physical incivilities**".

A fourth factor only loaded on presence of porches, decoration, and border. We called this factor "**decoration**".

Almost all items loaded high (≥ 0.4) on just one factor, except for sidewalk buffer (#31), sidewalk condition (#30/32), pedestrian oriented lighting (#36), and presence of pedestrian yield signs (#43_5), which each loaded on two factors. There were 15 items that did not load on any factor and hence, were excluded for the CFA: short segment (GIS), steep segment (GIS), type of front yard (#6), security warning sign (#10), industrial land (#13), agricultural land (#14), home business (#18), vacant or underdeveloped land (#19), graffiti (#29), footpath (#33), trees (#35), road oriented lighting (#36), neighborhood crime watch (#43_13), beware of dog or invisible fence signs (#43_15), and signs for cars regarding bike/pedestrian (combined index from #43_2, #43_6, and #43_7)).

The Cronbach's alpha and goodness-of-fit for the 1-factor models are presented in Table 4. The internal validity for these a posteriori constructs was high (range 0.43 to 0.73). The fit was acceptable according to the RMSEA, but according to the CFI and TLI the fit was slightly below the recommended cut-off values for some factors, but much higher compared to the a priori constructs. The models allowed for the following correlated errors: cul-de-sac and speed, visible children and adults, and buffer and sidewalk. The 4-factor model by urbanicity had worse fit than the separate 1-factor models, but allowed testing whether the factors were correlated after constraining some of the factor loadings to zero. The walkable neighborhood factor was negatively correlated with physical incivilities for both urban and rural segments (r = -0.12 and -0.55, respectively), and with the arterial/thoroughfare factor only in rural segments (r = -0.26). The parameter estimates for the hypothesized and derived constructs using the validation sample were not significantly different from those using the exploratory sample (Table 5), providing evidence of validity in our population.

**Table 4:** Standardized Cronbach's alpha and confirmatory factor analysis for constructs derived from EFA and Pearson correlation coefficients, exploratory sample (n = 6,388)

		**Standardized Cronbach's Alpha^§^**		**Goodness-of-fit**							
			
**Model**	**# items**	**Urban (n = 4,533)**	**Rural (n = 1,846)**	**Urban**				**Rural**			
					
				# parameters	CFI > 0.95	TLI > 0.95	RMSEA < 0.05	# parameters	CFI > 0.95	TLI > 0.95	RMSEA < 0.05
**1-factor**											
Arterial or thoroughfare	13	0.73	0.64	34	0.922	0.927	0.003	33	0.910	0.904	0.003
Walkable neighborhood	11	0.67	0.60	32	0.881	0.909	0.003	31	0.924	0.930	0.002
Physical incivilities	9	0.61	0.69	21	0.980	0.980	0.002	22	0.934	0.922	0.002
Decoration	3	0.43	0.48	9	1	1	0	9	1	1	0

**4-factor**	32	-	-	93	0.77	0.79	0.003	91	0.63	0.63	0.004
											
				
**Pearson correlations**				Arterial or toroughfare	Walkable nighborhood	Physical incivilities	Decoration	Arterial or thoroughfare	Walkable nighborhood	Physical incivilities	Decoration

Arterial or thoroughfare				1				1			
Walkable neighborhood				-0.07	1			-0.26	1		
Physical incivilities				0.25	-0.12	1		0.00	-0.55	1	
Decoration				-0.19	-0.24	-0.38	1	0.16	-0.07	-0.35	1

§Data are weighted by the road segment's length.

In the 4-factor model, there were 32 items instead of 36 because 4 items (#31 sidewalk buffer, #30/32 sidewalk condition, #36 pedestrian oriented lighting, and #43_5 presence of pedestrian yield signs) loaded on several factors.

Abbreviations: RMSEA root mean square error of approximation, CFI comparative fit index, and TLI Tucker-Lewis

*These are "rules of thumb" guidelines to interpret these indices (see paper for more information).

**Table 5:** Goodness-of-fit for multiple-group confirmatory factor analysis^§^: exploratory vs. validation samples^†^

	**Urban (n = 7,660)**					**Rural (n = 3,110)**				
		
**Model**	# parameters	CFI > 0.95	TLI > 0.95	RMSEA < 0.05	Difference test^‡^	# parameters	CFI > 0.95	TLI > 0.95	RMSEA < 0.05	Difference test^‡^
**1-factor**										
Arterial or thoroughfare	34	0.956	0.958	0.003	0.648	33	1.000	1.000	<0.001	0.972
Walkable neighborhood	32	0.953	0.953	0.002	0.743	31	0.888	0.888	0.003	0.549
Physical incivilities	21	0.983	0.984	0.001	0.669	22	0.985	0.984	0.003	0.855
Decoration	9	1.000	1.000	<0.001	0.852	9	1.000	1.000	<0.001	0.809

§ Data are weighted by the road segment's length.

† In urban areas: 7,660 road segments (4,537 and 3,123 in exploratory and validation subsamples). In rural areas: 3,110 road segments (1,851 and 1,259 in exploratory and validation samples).

‡ Ho: Invariance (i.e. all factor loadings, thresholds and correlated errors are the same) H1: Non invariance (i.e. different factor loadings, thresholds and correlated errors by sample).

Abbreviations: RMSEA root mean square error of approximation, CFI comparative fit index, and TLI Tucker-Lewis index

*These are "rules of thumb" guidelines to interpret these indices (see paper for more information).

Lastly, we explored 2-week test-retest reliability using Spearman correlation coefficients for 464 road segments (Table 6). The arterial or thoroughfare factor displayed near perfect reliability in both urban and rural segments (r = 0.96 for both). Both the physical incivilities factor (r = 0.77 to 0.78) and the walkable neighborhood factor (r = 0.79 to 0.82) had substantial to near perfect test-retest reliability in both urban and rural segments. The decoration factor displayed moderate reliability in urban segments (r = 0.50; 95% CI: 0.38–0.60) and lower reliability in rural segments (r = 0.39; 95% CI: 0.25–0.52).

**Table 6:** Two-week test-retest reliability using Spearman correlation coefficients with 95% confidence intervals (CI)

	**Spearman (95% CI)**	
	
**Construct**	**Urban (n = 255)**	**Rural (n = 209)**
	
Arterial or thoroughfare	0.96 (0.94, 0.97)	0.96 (0.95, 0.97)
Walkable neighborhood	0.79 (0.75, 0.84)	0.82 (0.77, 0.86)
Physical incivilities	0.78 (0.73, 0.82)	0.77 (0.71, 0.82)
Decoration	0.50 (0.38, 0.60)	0.39 (0.25, 0.52)

## Discussion

Using data collected from neighborhood audits and GIS-derived variables, we sought to confirm neighborhood constructs developed by others. The CFA analysis of the items that composed the a priori constructs physical incivilities, territoriality and social spaces from Caughy et al [8], and safety, aesthetics, destinations, and functionality from Pikora et al [19], indicated that the items composing a priori constructs did not hold in this geographic area with the exception of physical incivilities. Therefore we moved to EFA, where a four-factor solution was derived that included the following constructs: arterial or thoroughfare, walkable neighborhood, physical incivilities, and decoration. These constructs performed well in the CFA on the validation sample for both urban and rural road segments. Two-week test-retest reliability ranged from moderate to almost perfect for all except the decoration factor in rural areas.

### A priori Caughy et al Constructs

The three constructs from Caughy et al [8] (physical incivilities, territorality, and social spaces) were developed from a neighborhood audit conducted in Baltimore, Maryland. For the physical incivilities construct, we collected similar items to the original construct, with the addition of visible dogs. All of the a priori items loaded on both urban and rural segments, with the exception of the presence of visible dogs which was important in rural but not urban segments. When we moved to the EFA, the physical incivilities construct derived from the data included four of the original eight items, in addition to pedestrian oriented lighting and no trespassing sign. The physical incivilities construct appears empirically represented by these data. In prior research, physical incivilities was associated with increased levels of crime [27] and pregnancy-related behaviors [28].

The other two constructs from Caughy et al [8] did not hold in our data. For the territorality construct, we included similar items to the original index with the exception of two items. We did not collect whether residents reacted to the presence of raters, as rating was performed from a car rather than by walking. We also did not collect presence of security bars, as almost no homes had security bars in our study area. Similar to Laraia et al [18], we added several other items pertaining to signage when we explored the territorality construct. For the social spaces construct, we collected similar original items and expanded it to include presence of porches and sidewalks, also similar to Laraia et al [18].

These differences may be why the constructs did not hold in this geographic area. Both of these constructs (e.g., territorality and social spaces) were developed for use in the urban northeast US, where population density, park accessibility, and foot traffic patterns differ from the suburban and rural southeast US. Both constructs rely on specific types of indicators (e.g., short walls) and natural opportunities for social interactions (e.g., playgrounds) that were not as often present in our region. Documenting that these constructs do not function as expected is an important finding of this work, and suggests future development work in these areas. Empirically identifying items more specific to a latent construct shared across different types of neighborhoods for each study area may be an important undertaking not only to understand how the latent construct may manifest itself, but also as a data reduction technique that will minimize error.

### A priori Pikora et al Constructs

Considering the walking and bicycling framework by Pikora et al [19], the original instrument developed from this framework in Australia was titled SPACES [11] and was adapted to the US in other studies [29-32] to study walking and bicycling. Working from this instrument, we developed and modified items on the audit with applicability to rural and urban areas of central North Carolina. The adaptation of the SPACES instrument, such as revising questions, dropping items with low prevalence, may have been why the a priori constructs did not hold. Several items were dropped rather than modified from the original SPACES instrument due to measurement concerns, including whether the path formed a direct route or continuous route to destinations (functional construct), driveways and permanent obstructions in the path or lane (safety construct), and pollution (aesthetics construct). In addition, the original SPACES audit was conducted on foot, whereas our audit was conducted from a vehicle. Although the original constructs did not hold, many of the items mapped to newly derived data driven constructs. These changes to the instrument were done for use in our geographic area, but in turn may have compromised a direct test of replicability. It is possible that these original constructs may hold in geographic areas more similar to Perth, Australia where the tool was first developed.

Our analysis suggests some items that may be redundant or that did not contribute to any factor, including several secondary GIS measures (short road segment and steep segment) and a number of audit based measures including type of front yard (#6), security warning sign (#10), industrial land (#13), agricultural land (#14), home business (#18), vacant or underdeveloped land (#19), graffiti (#29), footpath (#33), trees (#35), road oriented lighting (#36), neighborhood crime watch (#43_13), beware of dog or invisible fence signs (#43_15), and signs for cars regarding bike/pedestrian (combined index from #43_2, #43_6, and #43_7). Several of these items were newly added, to capture features in more rural environments, but were found to not contribute to any underlying factor. However, they still may be important in other parts of the country and still may be predictive of physical activity as independent items.

Jago et al [30] also used a modified SPACES instrument to assess neighborhoods in Houston, Texas. Similar to our work, they dropped items with low variability and items that did not load on any factor. Using principal component analysis, they also dropped items that loaded on more than one factor. The remaining four data driven components accounted for 49% of the variance and included walking/cycling ease, tidiness, sidewalk characteristics, and street access/condition. These constructs also differed from the originally envisioned SPACES constructs, and the authors noted that this may be due to differences in measurement (self-reported ideas vs observed environmental data). These findings, together with our own, may indicate that a universal audit instrument may lose local heterogeneity.

### Considerations for Data Collection and Analysis

In the process of collecting, processing, and analyzing data using our PIN3 Neighborhood Audit data, several important lessons were learned that should be considered by others using this type of process. First, the factor analysis could not easily accommodate nominal variables with more than two levels. Thus, for questions such as condition of resident units, resident grounds, vacant/underdeveloped land, or public spaces, we added a "mixed condition" choice to describe conditions with extreme differences on the same road segment. While this may have better captured the characteristic of the road segment, it changed the previously ordinal variable to a nominal one. In many cases we had to collapse these variables into fewer categories. Future studies should consider whether nominal response options with more than two levels could be reworded to overcome this limitation of current software.

Second, the issues of missing data must be considered, whether due to incomplete answers or intended skip patterns. In our dataset, we had virtually complete data because we used a handheld device to collect the data. Where data were missing that was not due to a skip pattern, we treated it as missing at random. The PIN3 Neighborhood Audit tool included 7 intentional skips and in our analysis we either coded the missings to "not present" or treated the missings as another response option, depending on the specific variable. As these audit instruments evolve, consideration should be given to whether or not items with forced skip patterns are used. Intended skip patterns are acceptable, but at present the resultant variables will be nominal.

Third, consideration should be given to the type of rotation used in the factor analyses. Using orthogonal rotation forces the constructs to be statistically uncorrelated which is advantageous for subsequent use in statistical modeling as well as its simplicity and conceptual clarity [33]. In contrast, the oblique rotation allows factors to be statistically correlated. While this may better represent reality, since these neighborhood constructs may be intercorrelated, it also adds statistical complexity to future analyses. A priori, we had decided to derive the factors orthogonally because it simplified future analysis and this is what is presented in the results. However, we also derived correlated constructs using the Promax (oblique) rotation. The factor loadings were similar to those from Varimax (orthogonal) rotation, and Pearson correlations between constructs were low (range: -0.21, 0.16). Thus, using either an orthogonal or oblique rotation, our final data driven constructs were consistent.

### Limitations and Strengths

This study was conducted in four counties in central North Carolina and the road segments collected were based on where the PIN3 participants lived. This study included a large number of road segments (26% of all street segments in a 4-county area that included 7,150 miles of roads) with enough geographic diversity to explore urbanicity. However, our sample was neither random nor complete for the geographic area. Thus, it is not known how these results might generalize to other areas. While we expanded the use of the audit instrument to more rural areas, these segments were located within proximity to more urban areas; it is not known if results would change if this audit was conducted in more isolated rural geographies. Also, neighborhood observation represents a cross-sectional snapshot of the community and may miss neighborhood dynamics that change over time [8]. A strength of this study is that we collected test-retest reliability to further describe the measurement properties of our derived constructs. This study also collected items based on an a priori framework from Caughy et al [8] and SPACES [11].

## Conclusion

We demonstrated that several a priori theoretically derived constructs, developed in more urban areas, did not hold in our study area that included both urban and rural areas, with the exception of physical incivilities. Thus, we developed several data driven constructs using both directly observed neighborhood features and secondary GIS data. The results of our analyses yielded four reliably and objectively measured constructs that will be used to explore associations with physical activity. The usefulness of these factors is that they do not rely on self-report, they represent features of neighborhoods beyond existing census data, and they combine a number of items which may not be reasonably explored each separately on their own. Our work also extends these methods to more rural geographic areas. These constructs could be explored in other geographic areas to confirm their usefulness in other locations. Our protocol for confirmation of a priori factors and exploration of new factors when confirmation does not hold can be replicated by others.

## List of Abbreviations Used

CFA: confirmatory factor analysis; CFI: comparative fit index; EFA: exploratory factor analysis; GIS: geographic information systems; RMSEA: root mean square error of approximation; TLI: Tucker-Lewis index.

## Competing interests

The authors declare that they have no competing interests.

## Authors' contributions

KRE developed the aims of the study and drafted the paper, while all the remaining authors provided critical feedback to several earlier drafts of the paper. AHH assisted in the design of the study. DSA led the statistical analysis of the manuscript and helped write the methods and results sections, working with AHH and KRE on revisions. KRE, BAL, LM, and DAR helped develop and modify the audit instrument for this study. KRE oversaw audit data collection, in coordination with others, including LM. All authors read and approved the final manuscript.

## Supplementary Material

Additional file 1 **PIN3 Neighborhood Audit Instrument**. This file provides the questions used on the PIN3 Neighborhood Audit instrument. Click here for file

Additional file 2 **Descriptive statistics for GIS and PIN3 Neighborhood Audit variables for entire sample (n = 10,770)**. This file provides descriptive statistics for all of the GIS and neighborhood audit variables used in the analyses. Click here for file
