# Supplementary material for: Assessing urban and rural neighborhood characteristics using audit and GIS data: derivation and reliability of constructs
Source: Int J Behav Nutr Phys Act. 2009 Jul 20;6:44. doi: 10.1186/1479-5868-6-44 (PMC2726116; doi:10.1186/1479-5868-6-44)
Supplement: Additional file 1 — PIN3 Neighborhood Audit Instrument. This file provides the questions used on the PIN3 Neighborhood Audit instrument. [file 1479-5868-6-44-S1.pdf]

[Additional file 1] PIN3 Neighborhood Audit Instrument (Reference: Evenson et al, 2009, IJBNPA)

#	Item	Response Options
	Palm ID #	
	Rater ID #s	
	Date	
	Time of day	
	Street segment number	
	Street name	
**1**	Subjective assessment: Is this street walkable	Relative to other areas you have observed, please provide a subjective assessment.<br>☐ Strongly agree<br>☐ Agree<br>☐ Disagree<br>☐ Strongly disagree
**RESIDENTIAL LAND USE**		
**2**	Number of residential units	#<br>(**if 0, then skip entire section)**
**3**	Types of residential housing *(mark all that apply)*	☐ Single family - detached<br>☐ Multi-family/apartment/duplex<br>☐ Mobile homes/trailer home<br>☐ Housing authority/HUD projects<br>☐ New construction / renovation
**4**	Overall condition of most residential units	☐ Excellent condition<br>☐ Good condition<br>☐ Fair condition<br>☐ Poor/deteriorated condition<br>☐ Mixed condition *(extreme differences)*<br>☐ Cannot see because heavily wooded **(if cannot see, then skip entire section)**<br>☐ Cannot see for some other reason **(if cannot see, then skip entire section)**
**5**	Overall condition of resident-kept grounds	☐ Not applicable - no grounds<br>☐ Excellent condition<br>☐ Good condition<br>☐ Fair condition<br>☐ Poor/deteriorated condition<br>☐ Mixed condition *(extreme differences)*
**6**	Type of most front yards	☐ None<br>☐ Traditional lawn<br>☐ Landscaped<br>☐ Heavily wooded<br>☐ Mixed conditions *(extreme differences)*
**7**	Presence of porches	☐ None<br>☐ Less than half<br>☐ Half or more than half
**8**	Presence of some form of decoration	☐ None<br>☐ Less than half<br>☐ Half or more than half
**9**	Presence of border (fences/shrubs)	☐ None<br>☐ Less than half<br>☐ Half or more than half

**10**	Presence of visible security warning signs	☐ None<br>☐ Less than half<br>☐ Half or more than half
**11**	Any burned, boarded up, or abandoned residential units	☐ No<br>☐ Yes
**NONRESIDENTIAL LAND USE**		
**12**	Presence of nonresidential commercial (i.e., restaurants. cafes) land use	☐ None<br>☐ Yes, new construction / renovation<br>☐ Yes, existing<br>☐ Yes, new construction / renovation and existing
**13**	Presence of nonresidential industrial land use	☐ None<br>☐ Yes, new construction / renovation<br>☐ Yes, existing<br>☐ Yes, new construction / renovation and existing
**14**	Presence of nonresidential agricultural land	☐ No<br>☐ Yes, field or farm
**15**	Number of religious structures on segment	#
**16**	Overall condition of most buildings (commercial, industrial, agricultural, or religious)	☐ Not applicable<br>☐ Excellent condition<br>☐ Good condition<br>☐ Fair condition<br>☐ Poor/deteriorated condition<br>☐ Mixed condition *(extreme differences)*
**17**	Any burned, boarded up, or abandoned nonresidential units (commercial, industrial, agricultural, or religious)	☐ No<br>☐ Yes
**18**	Presence of home-based businesses (e.g., repairs, sales, etc)	☐ No<br>☐ Yes
**PUBLIC, RESIDENTIAL AND NONRESIDENTIAL SPACE / AESTHETICS**		
**19**	Presence of land that is vacant / underdeveloped	☐ None (**if none, skip to #21)**<br>☐ Less than half<br>☐ Half or more than half
**20**	Overall condition of land that is vacant / underdeveloped	☐ Excellent condition<br>☐ Good condition<br>☐ Fair condition<br>☐ Poor condition (illegal dumping)<br>☐ Mixed condition *(extreme differences)*
**21**	General condition of public spaces	☐ Excellent condition<br>☐ Good condition<br>☐ Fair condition<br>☐ Poor / deteriorated condition<br>☐ Mixed conditions *(extreme differences)*<br>☐ Not applicable (private road)

[Additional file 1] PIN3 Neighborhood Audit Instrument (Reference: Evenson et al, 2009, IJBNPA)

**22**	Visible people	☐ None (**skip to #24**)<br>☐ Children/youth only<br>☐ Adults only<br>☐ Children/youth and adults
**23**	Are the people being physically active	☐ No<br>☐ Yes, children/youth<br>☐ Yes, adults<br>☐ Yes, children/youth and adults
**24**	Any public or neighborhood park or playground in this segment *(mark all that apply)*	☐ No (**skip to #26**)<br>☐ Yes, park<br>☐ Yes, playground<br>☐ Yes, church park &/or playground
**25**	Overall condition of park and/or playground	☐ Excellent condition<br>☐ Good condition<br>☐ Fair condition<br>☐ Poor / deteriorated condition<br>☐ Mixed conditions *(extreme differences)*
**26**	Visible dogs	☐ No<br>☐ Yes, none are loose and out of yard<br>☐ Yes, at least one loose and out of yard
**27**	Amount of litter	☐ None (**skip to #29**)<br>☐ A little<br>☐ A moderate amount<br>☐ A considerable amount
**28**	Type of litter *(mark all that apply)*	☐ Nonalcoholic cans/bottles/paper<br>☐ Alcoholic cans/bottles<br>☐ Large items (tires, furniture, appliances, cars)<br>☐ Other litter
**29**	Amount of graffiti	☐ None<br>☐ A little<br>☐ A moderate amount<br>☐ A considerable amount
**WALKING AND BICYCLING AMENITIES**		
**30**	Presence of sidewalk	☐ None (**skip to #33**)<br>☐ One side of street, whole segment<br>☐ One side of street, partial segment<br>☐ Both sides of street, whole segment<br>☐ Both sides of street, partial segment
**31**	Sidewalk buffer	☐ Adjacent to street or curb (no buffer)<br>☐ Within 2 feet of street (buffer)<br>☐ Between 2-6 feet of street (buffer)<br>☐ Greater than 6 feet of street (buffer)
**32**	Sidewalk condition	☐ Good (very few bumps/cracks/ holes)<br>☐ Fair (some bumps / cracks / holes)<br>☐ Poor (many bumps / cracks / holes)<br>☐ Under repair
**33**	Presence of footpath along road	☐ None<br>☐ One side of street, entire length<br>☐ One side of street, partial length<br>☐ Both sides of street, entire length<br>☐ Both sides of street, partial length

**34**	Any trails that you can see in this segment	☐ No<br>☐ Yes, soft surface<br>☐ Yes, hard surface<br>☐ Both hard and soft surface
**35**	Trees shading walking area	☐ No trees along segment<br>☐ Yes, some trees along segment<br>☐ Yes, trees along entire segment
**36**	Public lighting	☐ None<br>☐ Road oriented<br>☐ Pedestrian oriented<br>☐ Both road and pedestrian oriented
**TRANSIT AND ROAD CHARACTERISTICS**		
**37**	Transit facilities (mark all that apply)	☐ None<br>☐ Bus stop without bench or shelter<br>☐ Bus stop with shelter<br>☐ Bus stop with bench
**38**	Number of lanes	Min number of lanes to cross: _____<br>Max number of lanes to cross: _____
**39**	Is road paved	☐ No<br>☐ Yes, paved only<br>☐ Both, paved and gravel
**40**	Highest speed limit for segment	MPH: # _______ (if unknown enter 0)
**41**	Presence of a shoulder or bike lane	☐ No<br>☐ Yes, soft surface<br>☐ Yes, hard surface but not a bike lane<br>☐ Yes, hard surface bike lane
**42**	On-street parking	☐ None or not allowed<br>☐ Allowed, but restricted<br>☐ Allowed, no restriction
**43**	Traffic control devices, crossing aids, and signs in segment (mark all that apply)	☐ None<br>☐ Traffic light(s)<br>☐ Flashing warning sign(s)<br>☐ Stop sign(s)<br>☐ Pavement marking / crosswalk(s)<br>☐ Yield to pedestrian paddles / signal / crossing street sign(s)<br>☐ "Share the road" bicycle sign<br>☐ Other pedestrian or bike friendly traffic signs<br>☐ Bicycle parking facilities<br>☐ Speed bumps<br>☐ Median / traffic island<br>☐ Curb extension(s)<br>☐ Neighborhood entrance signs<br>☐ Neighborhood crime watch<br>☐ No trespassing(s)<br>☐ Beware of dog / invisible fence<br>☐ Billboard

[Additional file 1] PIN3 Neighborhood Audit Instrument (Reference: Evenson et al, 2009, IJBNPA)

Acknowledgments:
The audit instrument was developed from:
(1) Caughy M, O'Campo P, and Patterson J. A brief observational measure for urban neighborhoods. Health Place 2001; 7:225-36.
(2) Pikora T, Bull F, Jamrozik K, Knuiman M, Giles-Corti B, Donovan R. Developing a reliable audit instrument to measure the physical environment for physical activity. Am J Prev Med 2002; 23:187-94.

For the development and modification of the instrument, we acknowledge (in alphabetical order): Kelly Evenson, Carrie Fesperman, Brian Frizzelle, Barbara Laraia, Annie Lux, Lynne Messer, and Daniel Rodriguez. The corresponding training materials were developed by (in alphabetical order): Kelly Evenson, Carrie Fesperman, Barbara Laraia, and Lynne Messer.
