# Supplementary material for: Assessing urban and rural neighborhood characteristics using audit and GIS data: derivation and reliability of constructs
Source: Int J Behav Nutr Phys Act. 2009 Jul 20;6:44. doi: 10.1186/1479-5868-6-44 (PMC2726116; doi:10.1186/1479-5868-6-44)
Supplement: Additional file 2 — Descriptive statistics for GIS and PIN3 Neighborhood Audit variables for entire sample (n = 10,770). This file provides descriptive statistics for all of the GIS and neighborhood audit variables used in the analyses. [file 1479-5868-6-44-S2.pdf]

[Additional File 2] Descriptive statistics for GIS and PIN3 Neighborhood Audit variables for entire sample, overall and by urbanicity (n=10,770)

Variable*	Frequency ‡ (# segments)			Weighted percent‡		
	Urban	Rural	Overall	Urban	Rural	Overall
Road segments	7,660	3,110	10,770	61.3	38.7	100.0
**GIS Variables**						
**Cul-de-sac or dead-end road segment**						
0 No	5,917	2,128	8,045	81.6	73.5	78.5
1 Yes	1,743	982	2,725	18.4	26.5	21.5
**Three or four way intersection**						
0 No	4,618	2,311	6,929	56.4	74.0	63.2
1 Yes	3,042	799	3,841	43.6	26.0	36.8
**Road segment length < 240m**						
0 No	1,497	1,121	2,618	44.6	71.0	54.8
1 Yes	6,163	1,989	8,152	55.4	29.0	45.2
**Speed limit**						
0: ≤ 25 MPH	5,679	2,180	7,859	68.7	56.7	64.0
1: 26 to ≤ 45 MPH	1,535	279	1,814	21.1	7.7	15.9
2: >45 MPH	446	651	1,097	10.2	35.6	20.0
**Half or more of the segment with a steep slope (>5%)**						
0 No	4,317	1,870	6,187	56.7	61.2	58.5
1 Yes	3,343	1,240	4,583	43.3	38.8	41.5
**Residential Land Use§**						
**1 Walkable street (subjective assessment)**						
1 Strongly agree	1,275	396	1,671	15.6	8.7	12.9
2 Agree	5,154	1,767	6,921	62.5	47.5	56.7
3 Disagree	830	585	1,415	12.4	25.2	17.3
4 Strongly disagree	399	359	758	9.6	18.7	13.1
**2 Residential units**						
0 No	1,449	556	2,005	18.8	15.7	17.6
1 Yes	6,211	2,554	8,765	81.2	84.3	82.4
**3 Types of residential housing**						
Single family-detached						
0 No	765	365	1,130	13.3	9.5	11.8
1 Yes	5,444	2,188	7,632	86.7	90.5	88.2
Multi-family/apartment/duplex						
0 No	4,890	2,323	7,213	76.2	91.9	82.4
1 Yes	1,319	230	1,549	23.8	9.2	17.6
Mobile homes/trailer home						
0 No	6,065	2,066	8,131	97.4	73.3	87.9
1 Yes	144	487	631	2.6	26.7	12.2
Housing authority/HUD projects						
0 No	6,185	2,546	8,731	99.6	99.8	99.7
1 Yes	24	7	31	0.4	0.2	0.3
New construction/renovation						
0 No	6,026	2,417	8,443	96.0	93.8	95.2
1 Yes	183	136	319	4.0	6.2	4.9
**4 Overall condition of most residential units§#**						
0 Excellent condition	2,219	1,092	3,311	32.5	36.3	33.9
1 Good condition	3,328	1,055	4,383	57.0	46.4	53.0
2 Fair, poor/deteriorated, or mixed condition	628	305	933	10.5	17.3	13.1

[Additional File 2] Descriptive statistics for GIS and PIN3 Neighborhood Audit variables for entire sample, overall and by urbanicity (n=10,770)

Variable*	Frequency ‡ (# segments)			Weighted percent‡		
	Urban	Rural	Overall	Urban	Rural	Overall
**5 Overall condition of residential-kept grounds§**						
0 Excellent condition	944	392	1,336	14.0	11.7	13.1
1 Good condition	3,547	1,394	4,941	61.7	63.7	62.5
2 Fair, poor/deteriorated, or mixed condition	1,303	533	1,836	24.3	24.6	24.4
**6 Traditional lawn or landscaped?§**						
0 No	422	156	578	8.1	6.9	7.6
1 Yes	5,753	2,295	8,048	91.9	93.1	92.4
**7 Porches§**						
0 None	267	123	390	3.8	2.9	3.5
1 Less than half	423	147	570	6.5	7.1	6.8
2 Half or more than half	5,485	2,181	7,666	89.7	90.0	89.8
**8 Some form of decoration§**						
0 None	331	127	458	4.9	3.8	4.5
1 Less than half	638	198	836	11.2	8.7	10.3
2 Half or more than half	5,205	2,127	7,332	83.9	87.4	85.2
**9 Border§**						
0 None	1,504	836	2,340	22.1	25.9	23.5
1 Less than half	2,393	998	3,391	43.5	51.0	46.3
2 Half or more than half	2,278	617	2,895	34.5	23.1	30.1
**10 Visible security warning signs§**						
0 None	2,259	1,344	3,603	34.3	50.7	40.6
1 Less than half	2,746	851	3,597	50.7	42.6	47.6
2 Half or more than half	1,170	257	1,427	15.0	6.6	11.8
**11 Any burned/boarded up/abandoned residential units§**						
0 No	5,994	2,331	8,325	96.3	91.2	94.4
1 Yes	181	121	302	3.7	8.8	5.6
**Nonresidential Land Use**						
**12 Commercial land use**						
0 No	6,934	2,872	9,806	87.3	90.7	88.7
1 Yes	726	237	963	12.7	9.3	11.3
**13 Industrial land use**						
0 No	7,562	3,060	10,622	98.0	97.0	97.6
1 Yes	98	49	147	2.0	3.0	2.4
**14 Agricultural land**						
0 No	7,628	2,877	10,505	99.2	80.9	92.1
1 Yes	32	232	264	0.8	19.1	7.9
**15 Religious structures**						
0 No	7,452	3,047	10,499	96.0	97.3	96.5
1 Yes	208	61	269	4.0	2.7	3.5
**16 Overall condition of most buildings**						
0 Not applicable	6,743	2,598	9,341	83.8	70.3	78.6
1 Excellent condition	253	150	403	5.1	8.0	6.2
2 Good condition	539	287	826	9.2	17.3	12.3
3 Fair condition	96	51	147	1.5	3.1	2.1
4 Poor/deteriorated condition	11	10	21	0.1	0.4	0.2
5 Mixed condition	15	13	28	0.3	1.0	0.6
**17 Any burned/boarded up/abandoned nonresidential units**						
0 No	7,585	3,052	10,637	98.5	97.0	97.9
1 Yes	75	56	131	1.5	3.0	2.1

[Additional File 2] Descriptive statistics for GIS and PIN3 Neighborhood Audit variables for entire sample, overall and by urbanicity (n=10,770)

Variable*		Frequency ‡ (# segments)			Weighted percent‡		
		Urban	Rural	Overall	Urban	Rural	Overall
**18**	**Home-based businesses**						
	0 No	7,564	3,051	10,615	98.3	95.9	97.3
	1 Yes	95	58	153	1.7	4.1	2.7
**Public, Residential, and Nonresidential Space / Aesthetics**							
**19**	**Vacant/underdeveloped land**						
	0 No	7,387	2,933	10,320	94.8	91.0	93.3
	1 Yes	273	177	450	5.2	9.0	6.7
**20**	**Overall condition of vacant/underdeveloped land**						
	1 Excellent condition	28	26	54	13.0	9.6	11.2
	2 Good condition	123	100	223	42.5	63.8	53.7
	3 Fair condition	108	49	157	40.9	25.8	33.0
	4 Poor condition	13	2	15	3.6	0.7	2.1
	5 Mixed condition	0	0	0	0.0	0.0	0.0
**21**	**General condition of public spaces &**						
	0 Excellent condition	1,749	1,030	2,779	23.5	31.8	26.6
	1 Good condition	4,911	1,574	6,485	65.7	55.0	61.6
	2 Fair, poor/deteriorated, or mixed condition	740	312	1,052	10.9	13.2	11.8
**22&23**	**Children/youth visible and physically active**						
	0 No visible children/youth	7,100	2,952	10,052	91.5	94.3	92.6
	1 Visible children/youth but not active	229	69	298	3.5	2.6	3.1
	2 Visible children/youth being active	325	85	410	5.1	3.2	4.3
**22&23**	**Adults visible and physically active**						
	0 No visible adults	5,126	2,359	7,485	61.8	71.7	65.6
	1 Visible adults but not active	1,284	505	1,789	19.1	19.0	19.0
	2 Visible adults being active	1,243	241	1,484	19.2	9.3	15.4
**24**	**Any public or neighborhood park or playground§**						
	0 No	7,259	2,978	10,237	90.8	95.2	92.5
	1 Yes	401	130	531	9.2	4.8	7.5
**25**	**Overall condition of park and/or playground**						
	1 Excellent condition	160	70	230	44.8	53.8	47.1
	2 Good condition	200	43	243	46.4	32.9	43.0
	3 Fair condition	29	11	40	5.3	8.5	6.1
	4 Poor/deteriorated condition	8	6	14	2.9	4.8	3.4
	5 Mixed condition	3	0	3	0.6	0.0	0.4
**26**	**Visible dogs**						
	0 No	7,018	2,797	9,815	90.2	85.0	88.2
	1 Yes	642	312	954	9.8	15.0	11.8
**27**	**Amount of litter**						
	0 None	3,682	1,610	5,292	40.6	36.4	38.9
	1 A little	3,582	1,329	4,911	52.2	53.6	52.7
	2 A moderate or considerable amount	396	171	567	7.3	10.0	8.3
**28**	**Type of litter**						
	Nonalcoholic cans/bottles/paper						
	0 No	96	63	159	2.4	3.6	2.8
	1 Yes	3,881	1,436	5,317	97.6	96.4	97.2
	Alcoholic cans/bottles						
	0 No	3,361	1,176	4,537	81.4	68.0	76.0
	1 Yes	616	323	939	18.6	32.0	24.0
	Large items						

[Additional File 2] Descriptive statistics for GIS and PIN3 Neighborhood Audit variables for entire sample, overall and by urbanicity (n=10,770)

Variable*	Frequency ‡ (# segments)			Weighted percent‡		
	Urban	Rural	Overall	Urban	Rural	Overall
0 No	3,755	1,328	5,083	91.9	88.0	90.4
1 Yes	222	171	393	8.1	12.0	9.6
Other litter						
0 No	3,709	1,335	5,044	91.4	86.9	89.6
1 Yes	268	164	432	8.6	13.1	10.4
**29 Graffiti**						
0 No	7,406	3,060	10,466	96.0	98.1	96.8
1 Yes	252	50	302	4.0	1.9	3.2
**Walking and Bicycling Amenities**						
**30&32 Sidewalk condition**						
0 No sidewalk	4,791	2,604	7,395	61.6	88.4	72.0
1 One or two sides in fair/poor condition or under repair	603	18	621	7.3	0.4	4.6
2 One side in good condition	1,509	377	1,886	19.4	8.5	15.2
3 Both sides in good condition	756	111	867	11.7	2.7	8.2
**31 Sidewalk buffer**						
0 No sidewalk	4,791	2,604	7,395	61.6	88.4	72.0
1 Adjacent to street or curb (no buffer)	677	115	792	9.2	2.7	6.7
2 Buffer within 2 feet of street	1,129	217	1,346	14.2	4.3	10.4
3 Buffer more than 2 feet of street	1,063	174	1,237	15.0	4.6	11.0
**33 Footpath along road**						
0 No	7,419	3,071	10,490	96.4	98.5	97.2
1 Yes	240	38	278	3.6	1.5	2.8
**34 Any trails that you can see in this segment**						
0 No	7,176	2,829	10,005	89.9	82.6	87.0
1 Yes, only soft surface	290	234	524	6.7	15.9	10.3
2 Yes, hard surface or both hard and soft surface	193	47	240	3.4	1.5	2.7
**35 Trees shading walking area**						
0 No trees along segment	4,853	2,308	7,161	61.4	70.7	65.0
1 Yes, some trees along segment	2,162	595	2,757	30.1	22.1	27.0
2 Yes, trees along entire segment	645	207	852	8.5	7.1	8.0
**36 Road oriented public lighting**						
0 No	2,402	2,197	4,599	28.8	76.9	47.4
1 Yes	5,258	912	6,170	71.2	23.1	52.6
**36 Pedestrian oriented public lighting**						
0 No	6,940	2,718	9,658	90.5	91.6	90.9
1 Yes	720	391	1,111	9.5	8.4	9.1
**Transit and Road Characteristics**						
**37 Bus facilities**						
0 No	7,131	3,088	10,219	91.5	99.4	94.6
1 Yes	522	19	541	8.5	0.6	5.4
**38 Number of lanes**						
0 One lane to cross maximum	252	295	547	2.7	9.0	5.1
1 Two lanes to cross maximum	6,571	2,564	9,135	81.0	80.5	80.8
2 Three or more lanes to cross maximum	837	250	1,087	16.3	10.5	14.1
**39 Road paved**						
0 No	456	543	999	5.7	19.1	10.9
1 Yes	7,204	2,566	9,770	94.3	80.9	89.1
**40 Highest speed limit sign on segment**						
0: ≤ 25 MPH	1,636	315	1,951	61.2	32.0	51.3

[Additional File 2] Descriptive statistics for GIS and PIN3 Neighborhood Audit variables for entire sample, overall and by urbanicity (n=10,770)

Variable*	Frequency ‡ (# segments)			Weighted percent‡		
	Urban	Rural	Overall	Urban	Rural	Overall
1: 26 to ≤ 45 MPH	717	393	1,110	34.2	57.2	42.0
2: >45 MPH	26	34	60	4.6	10.9	6.7
**41 Shoulder or bike lane**						
0 No	7,232	2,906	10,138	90.5	89.7	90.2
1 Yes	427	203	630	9.5	10.3	9.8
**42 On-street parking**						
0 No	5,107	2,737	7,844	66.4	90.7	75.8
1 Yes	2,552	372	2,924	33.6	9.3	24.2
**43_4 Pavement markings, crosswalk**						
0 No	7,033	3,055	10,088	90.9	98.6	93.8
1 Yes	606	41	647	9.1	1.4	6.2
**43_5 Yield to pedestrian paddles, signal, crossing street sign**						
0 No	7,257	3,041	10,298	93.6	97.9	95.3
1 Yes	382	55	437	6.4	2.1	4.7
**43_8 Bicycle parking facilities**						
0 No	7,594	3,093	10,687	99.1	99.9	99.4
1 Yes	45	3	48	0.9	0.1	0.6
**43_12 Neighborhood entrance sign**						
0 No	7,151	2,857	10,008	91.2	92.3	91.6
1 Yes	488	239	727	8.8	7.7	8.4
**43_13 Neighborhood crime watch**						
0 No	7,037	2,898	9,935	90.3	91.1	90.6
1 Yes	602	198	800	9.7	8.9	9.4
**43_14 No trespassing sign**						
0 No	6,924	2,630	9,554	86.7	72.1	81.0
1 Yes	715	466	1,181	13.3	27.9	19.0
**43_15 Beware of dog, invisible fence**						
0 No	6,988	2,808	9,796	89.0	85.9	87.8
1 Yes	651	288	939	11.0	14.1	12.2
**43_16 Billboard**						
0 No	7,568	3,010	10,578	98.1	95.1	96.9
1 Yes	71	86	157	1.9	4.9	3.1
**43† Control devices oriented for cars**						
0 None	2,950	1,726	4,676	33.9	54.1	41.7
1 One	4,253	1,224	5,477	56.0	40.4	50.0
2 Two to five	436	146	582	10.1	5.5	8.3
**43€ Signs for cars about bike/pedestrian**						
0 No	7,181	2,948	10,129	91.6	92.4	91.9
1 Yes	479	162	641	8.4	7.6	8.1

*The item number corresponds to the question number in supplementary file #1.

‡ Weighted by the road segment's length. Sample sizes may not add to 10,770 due to missing values or skips.

§ Non residential segments were left as missing.

136 segments could not be determined due to woods or other reasons, so they were set to missing

& 453 segments were coded as "not applicable (private road)" and were set to missing

† Derived as a count for presence of traffic lights, stop signs, speed bumps, median/traffic islands, and curb extensions (#43_1, 43_3, 43_9, 43_10, and 43_11).

€ Presence of either a flashing warning sign, "share the road" bicycle sign or other pedestrian or bike friendly traffic signs (#43_2, 43_6, and 43_7).

Item #1, 3, 16, 20, 25, 28, 40, 43_8, and 43_16 were not used in the analyses. Item #2 was used to indicate a skip pattern.
